# Development of an integrated 200K SNP genotyping array and application for genetic mapping, genome assembly improvement and genome wide association studies in pear (*Pyrus*)

**DOI:** 10.1111/pbi.13085

**Published:** 2019-02-17

**Authors:** Xiaolong Li, Jugpreet Singh, Mengfan Qin, Siwei Li, Xun Zhang, Mingyue Zhang, Awais Khan, Shaoling Zhang, Jun Wu

**Affiliations:** ^1^ Centre of Pear Engineering Technology Research State Key Laboratory of Crop Genetics and Germplasm Enhancement Nanjing Agricultural University Nanjing China; ^2^ Plant Pathology and Plant‐Microbe Biology Section Cornell University Geneva NY USA

**Keywords:** *Pyrus*, SNP array, genotyping, genetic map, genome assembly improvement, genome wide association studies

## Abstract

Pear (*Pyrus*; 2*n* = 34), the third most important temperate fruit crop, has great nutritional and economic value. Despite the availability of many genomic resources in pear, it is challenging to genotype novel germplasm resources and breeding progeny in a timely and cost‐effective manner. Genotyping arrays can provide fast, efficient and high‐throughput genetic characterization of diverse germplasm, genetic mapping and breeding populations. We present here 200K AXIOM ^®^ PyrSNP, a large‐scale single nucleotide polymorphism (SNP) genotyping array to facilitate genotyping of *Pyrus* species. A diverse panel of 113 re‐sequenced pear genotypes was used to discover SNPs to promote increased adoption of the array. A set of 188 diverse accessions and an F_1_ population of 98 individuals from ‘Cuiguan’ × ‘Starkrimson’ was genotyped with the array to assess its effectiveness. A large majority of SNPs (166 335 or 83%) are of high quality. The high density and uniform distribution of the array SNPs facilitated prediction of centromeric regions on 17 pear chromosomes, and significantly improved the genome assembly from 75.5% to 81.4% based on genetic mapping. Identification of a gene associated with flowering time and candidate genes linked to size of fruit core *via* genome wide association studies showed the usefulness of the array in pear genetic research. The newly developed high‐density SNP array presents an important tool for rapid and high‐throughput genotyping in pear for genetic map construction, QTL identification and genomic selection.

## Introduction

The development and application of molecular markers in plant genetics and breeding has seen great progress in recent years (Rasheed *et al*., 2017). Restriction fragment length polymorphisms (RFLPs) were among the earliest types of molecular markers (Tanksley *et al*., 1989), followed by the development of other commonly used DNA markers like random amplified polymorphic DNA (RAPDs), sequence‐characterized amplified regions (SCARs), simple sequence repeats (SSRs) and SNPs (Chagné *et al*., 2008; Paran and Michelmore, 1993; Tautz and Renz, 1984; Williams *et al*., 1990). The frequent occurrence of single nucleotide polymorphisms (SNPs) across the genomes of plant species makes them most suitable for genetic discovery research and marker‐assisted breeding (Bevan and Uauy, 2013; Varshney *et al*., 2009). SNP markers have been widely utilized in genetic diversity studies, high density genetic linkage map constructions and identification of loci or genes associated with the complex traits in many plant species (Chen *et al*., 2014; Khan *et al*., 2012; Mccouch *et al*., 2002; Unterseer *et al*., 2014; Winfield *et al*., 2016). Cost reduction and increased throughput of next‐generation sequencing (NGS) technologies have recently facilitated large‐scale discovery of genome‐wide SNP markers. Although sequencing‐based technologies have several advantages in marker discovery and development, repeated screening of new and large plant populations is still expensive and analytically challenging. SNP‐based chips or arrays, once developed, can significantly reduce time and cost of genotyping new populations for efficient plant breeding (Rasheed *et al*., 2017). SNP arrays have been increasingly adopted for genome‐wide genotyping, QTL mapping, genome wide association studies (GWAS) and genomic selection in both model and non‐model plant species like maize (Unterseer *et al*., 2014), wheat (Winfield *et al*., 2016), rice (Chen *et al*., 2014; Yu *et al*., 2014), barley (Bayer *et al*., 2017), soybean (Lee *et al*., 2015; Wang *et al*., 2015), tomato (Sim *et al*. 2012), potato (Vos *et al*., 2015) and peanut (Clevenger *et al*., 2017).

Developing a dense marker array for highly heterozygous fruit crops is always challenging due to the genome complexity and rapid linkage disequilibrium (LD) decay (Evans *et al*., 2014; Unterseer *et al*., 2014; Wu *et al*., 2014; Yamamoto *et al*., 2014; Yao *et al*., 2017). At present, SNP arrays have been developed for some highly heterozygous species like apple (Bianco *et al*., 2014, 2016), peach (Verde *et al*., 2012), strawberry (Bassil *et al*., 2015), diploid Sweet Cherry and tetraploid Sour Cherry (Peace *et al*., 2012) and grape (Laucou *et al*., 2018). The Apple480K array is currently the largest SNP array in a fruit tree species. It captures the genetic diversity in cultivated apple, with an ascertainment bias towards *Malus* × *domestica* (Bianco *et al*., 2016). A 9K SNP array is also available for peach. This array has a limited number of unevenly distributed SNPs across the peach genome with adjacent marker gaps up to 1254 kb for some genomic regions (Verde *et al*., 2012). Similar marker arrays with a limited number of markers have been developed in other Rosaceae crops as well. A 6K SNP array was developed and evaluated for diploid sweet cherry and tetraploid sour cherry (Peace *et al*., 2012). The Affymetrix IStraw90 Axiom array was developed using the integration of SNP and InDels from one diploid and 19 octoploid accessions of strawberry (Bassil *et al*., 2015). These efforts led by the RosBREED community (Iezzoni *et al*., 2016) and FruitBreedomics project (Laurens *et al*.,^2018^), have provided useful array resources to study genome structure and diversity analysis and to perform QTL mapping and GWAS (Urrestarazu *et al*., 2017) for important economic traits in the respective fruit species. In some of these arrays, the limited number of SNPs and germplasm diversity potentially restrict their broader utilization in molecular breeding applications. Ideally, a marker array should have genome‐wide distribution of markers at high density from the diverse gene pool of a particular species. Genome resequencing of diverse accessions can significantly improve the marker density for array development in highly heterozygous fruit tree species (Bianco *et al*., 2016; Wu *et al*., 2018).

Pear (*Pyrus*) is an economically important temperate rosaceous fruit crop, with an annual worldwide production of ~27 million tons (FAOSTAT, 2016, http://www.fao.org/faostat/en/#home). It has a small genome size of 527 Mb with approximately 53.1% (271.9 Mb) repetitive sequences (Wu *et al*., 2013). Its high heterozygosity (~1.02%), rapid LD decay, self‐incompatibility, clonal propagation and a long generation cycle, require a large number of evenly distributed markers across the entire genome for identification of minor effect QTLs and fine mapping of QTL regions (Wu *et al*., 2018). Various types of molecular markers have been developed in pear for genetic mapping of commercially important traits. For instance, the first genetic maps were constructed using a limited number of markers, including RAPD (Iketani *et al*., 2001), amplified fragment length polymorphism (AFLP) and SSR (Liu *et al*., 2010; Terakami *et al*., 2009) in Asian pear. Another low‐density linkage map of Japanese pear (*Pyrus pyrifolia* Nakai) was constructed using 335 SSRs and AFLP markers. Recently, high‐density genetic maps were constructed using SNP and SSR markers for mapping several fruit quality traits in Asian pear (Wu *et al*., 2014) and to anchor the genome sequence of ‘Bartlett’ (*Pyrus communis*, cultivated European pear) for synteny analysis between European and Asian pear (Li *et al*., 2017). A dense genotyping array can be used to identify shared markers and integrate linkage maps of different pear species for comparative genetics analysis. Development of high‐density genetic maps can provide useful markers to integrate unanchored scaffolds for improvement of genome assembly. In addition, an integrated 9K SNP array of 1096 SNP markers from three European pear (*P. communis* L.) cultivars and 7692 SNPs from the IRSC apple Infinium^®^ II 8K array have demonstrated marker transferability across Maloideae species (Montanari *et al*., 2013).

There is a wide range of variability for morphological, physiological and genetic traits in a worldwide germplasm of 22 well recognized wild and cultivated species and more than 5000 accessions for pear (Bell *et al*., 1996). Although availability of diverse germplasm is ideal for the application of GWAS to identify QTLs, there are only a few attempts at GWAS in pear. For example, (Kumar *et al*., 2017) performed GWAS to identify marker‐trait associations for fruit colour, fruit shape and fruit quality traits in 214 pear accessions. In general, the low number of available markers in addition to complex genetics has noticeably restricted the application of GWAS for the identification of functional alleles in pear germplasm. Here, we report the development and validation of a 200K SNP Affymetrix Axion^®^ genotyping array for wild and cultivated types from the Asian and European genepools. We further discuss potential applications of the array. The 200K AXIOM^®^ PyrSNP array is currently the first comprehensive SNP array in pear and has been commercially released for broader use in bi‐parental QTL mapping, GWAS and genomic selection in pear breeding.

## Results

### Sequence alignment, SNP detection and selection

**Figure 1:**
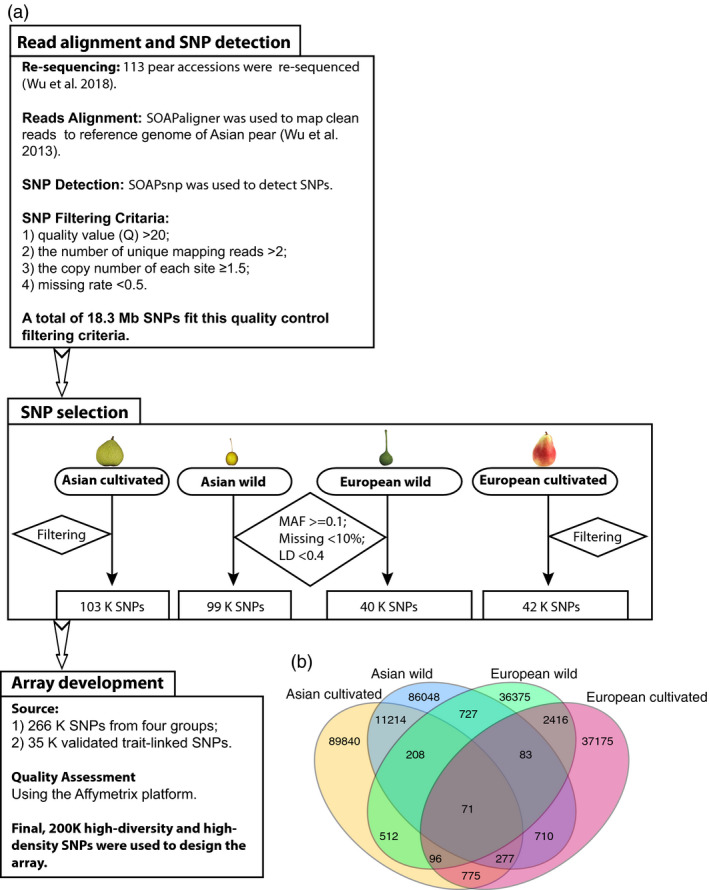
Single nucleotide polymorphism (SNP) detection and selection workflow. (a) The SNPs were selected based on four different populations, and then the same quality filtering was used to filter the SNPs. Finally, 200K SNP markers from the 18.3 M SNPs, including the validated trait‐link SNPs, were incorporated into the 200K AXIOM ^®^ PyrSNP array. (b) The Venn diagram of SNPs integrated into the 200K pear array. Light yellow represents the SNP set from Asian cultivated group; Light red represents the European cultivated group; Light blue represents the Asian wild group; Light green the European wild group.

Genome re‐sequencing of 113 pear accessions (*Pyrus*) (Table S1) with detailed methods for sequence alignment and SNP detection have been described by Wu *et al*. (2018). A total of 18.3 million high‐quality variants have been identified in the pear genome. We considered the genome‐wide uniform distribution and location of SNPs on or close to genes as equally important factors for SNP selection. A set of 200 481 SNPs (Figure S1; Table S2) uniformly distributed across the genome and in genic regions of the pear genome was selected from the original 18.3 million SNPs to develop the 200K AXIOM^®^ PyrSNP array. A detailed description about the detection, filtering and final selection of SNPs included in the array has been provided in Methods and Figure 1a. First, various sets of markers were identified in the four groups, namely 99 338 SNPs in Asian wild, 102 993 SNPs in Asian cultivated, 40 448 SNPs in European wild, and 41 603 SNPs in European cultivated. A total of 17 895 SNPS were common among all possible pairs in the four groups, giving a first set of 266 527 SNPs. After Quality assessment using the Affymetrix platform, a final set of 200 481 SNPs was retained for the Axiom array. We found that more SNPs were common within Asian groups and European groups, than between Asian and European groups (Figure 1b). The highest number (11 770) of common SNPs was present between Asian cultivated and wild group, compared to 2666 SNPs between European cultivated and wild group. A total of 1089 SNPs was common between Asian and European wild group, and 1219 SNPs were common between Asian and European cultivated group. Only 71 SNPs were common between all four groups.

### Validation of the PyrSNP array

**Figure 2:**
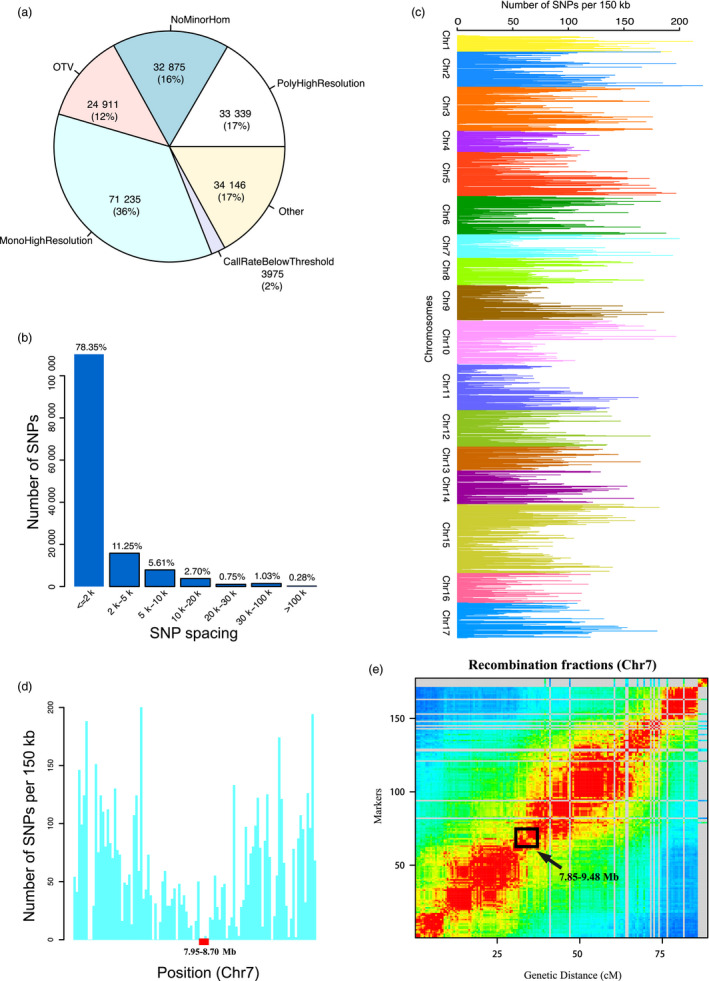
Summary of the informativeness of the converted SNPs. (a) The SNPs in the 200K AXIOM ^®^ PyrSNP array were classified into six types. (b) The distribution of inter‐SNP spacing. (c) The distribution of the converted SNPs on the array in sliding 150 kb windows along 17 pear chromosomes. (d) The distribution of the converted SNPs on the array in sliding 150 kb windows on chromosome 7. The red dot represents the candidate position of centromere. (e) The recombination fractions along the chromosome 7. *x*‐Axis indicated the genetic distance. Red represented the high recombination level; blue represented the low recombination level.

In the validation of the 200K AXIOM^®^ PyrSNP array with 188 diverse pear accessions (Table S3) and 98 F_1_ individuals, all samples passed the quality assessment with a high DQC value (>0.89) and call rate (>95%). Use of duplicated samples identified 99.4% SNP reproducibility. The SNP genotyping results from the 286 accessions were initially classified into six categories based on SNP quality control metrics (http://www.affymetrix.com). Only 17% of the SNPs (33 339 of 200 481 SNPs) could be converted based on the most stringent criteria described in method section (Figure 2a). Upon disregarding the filtering parameters Fisher's linear discriminant (FLD), HetSO and HomRO and only using CR ≥95% as the filtering option, about 93.9% (*n* = 188 346) of the total array SNPs were converted. Moreover, approximately 17% of the SNPs on the array that belonged to the ‘Other’ category were filtered out due to below‐threshold quality of genotypes. The remaining 166 335 high‐quality SNPs, including 140 632 SNPs on 17 chromosomes and 25 703 SNPs on unanchored scaffold sequences, provided the most reliable genotypes and were used for further analyses.

### Features of the PyrSNP array

Across 17 pear chromosomes, 140 632 high‐quality SNPs were distributed, with an average spacing of 3.6 kb. About 95.22% of the adjacent SNP pairs were spaced at <10 kb, whereas 78.35% were shorter than 2 kb and 11.25% were located within 2–5 kb (Figure 2b). The spacing between 393 SNP pairs exceeds more than 100 kb (Figure 2b), with greatest spacing of about 867 kb. Despite these few large gaps, the remaining SNPs were uniformly distributed across the pear genome. SNP count, distribution patterns and density across the pear genome were analyzed using a 150 kb sliding window to predict centromeric regions on pear chromosomes. The lowest SNP count was observed in the central part of the chromosomes, while telomeres had relatively more SNPs (Figure 2c; Figure S3). Telomere regions of chromosomes are enriched in genes, while centromeric regions are gene‐poor and repeat element‐rich regions in many plant species (Lee *et al*., 2013; Chen *et al*., 2002; Saintenac *et al*., 2009; Schnable *et al*., 2009). The chromosomal distribution of SNPs on the 200K AXIOM^®^ PyrSNP array corresponds with gene richness along the chromosomes. Thus, based on these, SNP distribution helped to propose putative centromeric regions for each of the pear chromosomes (Figure S3). For instance, based on SNP density at the terminal and central parts of chromosome 7, the candidate position of centromeric region was predicted to be located on 7.95–8.70 Mb of chromosome 7 (0.75 Mb in length) (Figure 2d). We have calculated recombination fractions across the entire pear genome by constructing a genetic linkage map of ‘Cuiguan’ × ‘Starkrimson’ bi‐parental population. A decrease of recombination fraction was observed at the position 7.85–9.48 Mb (1.63 Mb in length) of chromosome 7 (Figure 2e), which aligned with the centromeric region detected above. Comparison of 140 632 SNPs against the annotated ‘Dangshansuli’ (http://peargenome.njau.edu.cn) genome showed that about 85% (*n* = 119 622) of these SNPs were present in the 23 006 genes or their 2 kb flanking promoter regions. Similarly, about 68% (17 362 out of 25 703) of SNPs located in scaffolds were mapped to 2383 predicted genes. Overall, the converted SNPs covered about 60% of the annotated genes (42 341) in ‘Dangshansuli’ genome (http://peargenome.njau.edu.cn). Further annotation of these SNPs showed that 9.39% (12 869) of the SNPs (136 984) in genic and promoter regions were non‐synonymous sites, 25.4% were synonymous sites and 65.62% were located in intronic region of genes. Among 12 869 non‐synonymous sites, we identified 240 SNPs involved in early termination or elongation of the transcripts.

### Applications of the PyrSNP array

#### Assessment of the population genetic structure of diverse pear accessions

**Figure 3:**
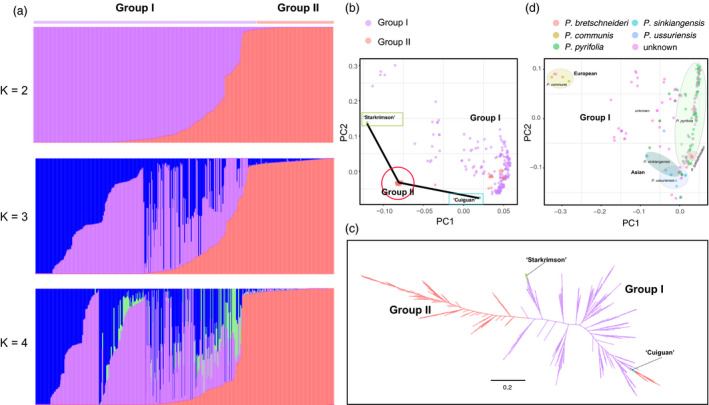
Population structure of 286 pear accessions. (a) Population structure analysis of 286 accessions. The numbers of clusters (*K*) were set from 2–4. Each colour represents a single population. Each bar represents one accession and different coloured segment represents the proportion from ancestral populations. (b) PCA of 286 pear accessions. The same colour represents an individual from the same group. (c) A maximum‐likelihood tree of 286 pear accessions. (d) PCA of 188 pear accessions. The same colour represents an individual from the same species.

Genetic diversity, relationships and clustering of diverse pear accessions were assessed by population structure analysis based on non‐negative matrix factorization (SNMF) algorithms, principal component analysis (PCA) and a maximum‐likelihood (ML) tree construction. Population structure analysis using *K* = 2 separated the 286 pear accessions into two groups: Group I from the diverse germplasm and Group II from the hybrid F_1_ progeny of ‘Cuiguan’ (*P. pyrifolia*, cultivated Asian pear) × ‘Starkrimson’ (*P. communis*, cultivated European pear) (Figure 3a). With subsequent increase in defined cluster number (*K* = 3–4), new groups split from group I showing admixture in the population. Group II still remained isolated during the *K* = 2–4 increase. These results suggest that Group I exhibit higher genetic diversity in comparison to Group II; most likely due to bi‐parental nature of the latter population. Similar results were obtained from PCA (Figure 3b) and phylogenetic analysis (Figure 3c). PCA analysis also showed that Group I had diverse pear accessions. In addition, except few F1 progeny of ‘Cuiguan’ × ‘Starkrimson’, grouping closer to the female parent ‘Cuiguan’, all other individuals in F_1_ hybrid progeny were clustered together to constitute Group II with approximately equal distance from the female parent ‘Cuiguan’ and the male parent ‘Starkrimson’ in the PCA biplot. PCA could clearly separate *P*. *pyrifolia*,* P. bretschneideri*,* P. sinkiangensis* and *P. ussuriensis* from each other, especially European pear species (*P. communis*), which were far away from Asian pear species (Figure 3d). Population structure also showed that Asian pears hold the higher genetic diversity, comparing to European pear in our study (Figure S2). Combined with Figure 3b, we also observed that ‘Cuiguan’, a *P. pyrifolia* species, grouped closer to Asian group, while ‘Starkrimson’, a *P. communis* species, closer to European group, suggesting reliability of relationship and clustering of Group I. These results also helped with speculation on the identities of ‘unknown’ accessions. For example, two samples with unknown identity clustered into European group (*P. communis*), indicating that they belong to or are more closely related to European pear species (Figure 3d).

#### High‐density genetic map construction and improvement of pear genome assembly

**Figure 4:**
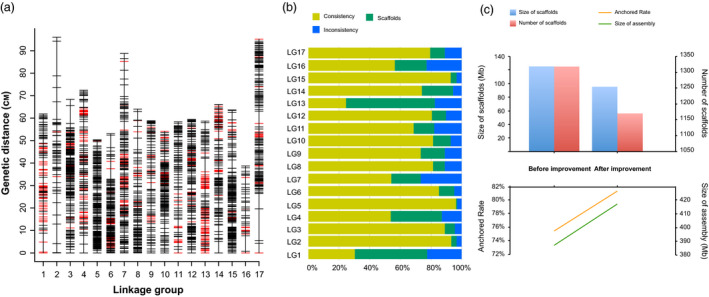
Construction of genetic maps to improve the genome assembly. (a) Distribution of 2388 SNP markers on 17 Linkage groups. A black bar indicates a SNP marker, and a red bar indicates a marker on the scaffolds. Linkage group number is shown on the *x*‐axis and genetic distance (cM) is shown on the *y*‐axis. (b) The consistency between the order of markers on linkage map and genome sequence assembly. The yellow bar represents the rate of consistency; blue bar the rate of inconsistency; green bar the rate anchored to the scaffolds. (c) A comparison before and after improving genome assembly. The blue bar represents the size of unanchored scaffolds; the red bar represents the number of unanchored scaffolds; the yellow line represents the anchored rate; the green line represents the size of assembly.

Parental genotypes ‘Cuiguan’, ‘Starkrimson’ and their F1 hybrid progeny of 98 individuals were genotyped using 200K AXIOM^®^ PyrSNP array to construct genetic map and to improve Asian pear genome assembly. After filtering for quality and missing data, a total of 7173 high‐quality including 6083 and 1090 SNPs across 17 pear chromosomes and unanchored scaffolds respectively, were polymorphic in this population. At LOD score >4.0, 2388 SNPs out of 7173 SNP markers were grouped and ordered to obtain 17 linkage groups (LGs) (Figure 4a; Table 1). Details of the genetic map are provided in Table S4. The genetic linkage map spans 1108 cM with average genetic distance of 0.46 cM. The maximal gap was observed on LG2 with 15.4 cM, and an average largest marker interval across 17 chromosomes was 5.7 cM (Table 1). LG3 contained highest 229 markers whereas lowest number of markers were present on LG16 (*n* = 39 markers). The consistency between the order of markers on linkage map and genome sequence assembly was 71% on average across 17 LGs (Figure 4b; Table S5). A set of 355 SNPs on 146 unanchored scaffolds (11.1% of 1318 scaffolds in pear genome) that represents a total of 30 Mb, were mapped to either of the 17 LGs (Figure 4c). The genetic map positions of these SNPs helped incorporate the unanchored scaffold into 17 pear chromosomes (Table S6) and improved the genome assembly from 75.5% to 81.4% (Figure 4c). For example, five SNPs on the scaffold132.0.1 were all mapped to LG1 and have been considered as a part of chromosome 1 in the current pear genome assembly.

**Table 1:** Summary of integrated pear linkage groups based on a segregating 98 F1 progeny of ‘Cuiguan’ × ‘Starkrimson’

Linkage group	No. of SNPs	Length (cM)	Max gap (cM)	Average interval (cM)
LG1	89	61.9	4.4	0.70
LG2	58	96.0	15.4	1.66
LG3	229	68.4	7.9	0.30
LG4	138	72.4	4.0	0.52
LG5	194	50.4	1.8	0.26
LG6	208	53.1	5.6	0.26
LG7	177	88.8	5.2	0.50
LG8	117	64.0	5.2	0.55
LG9	90	58.8	3.0	0.65
LG10	151	54.1	2.3	0.36
LG11	83	58.4	3.2	0.70
LG12	179	59.4	2.2	0.33
LG13	115	58.7	6.1	0.51
LG14	124	66.1	5.2	0.53
LG15	221	63.7	3.7	0.29
LG16	39	38.7	6.9	0.99
LG17	176	95.1	14.2	0.54
Total	2388	1108	5.7	0.46

#### Genome‐wide association study of phenological and fruit traits in pear

Statistical analysis showed significant phenotypic variation in 18 traits across 219 pear accessions (Table S7). These traits exhibited moderate to high broad‐sense heritability (Table 2), but the values could be over‐estimated due to a ‘common environment’ effect for the considered replicates (i.e. several fruits collected on a single tree). Mostly, traits were normally distributed except for early bloom, full bloom and fall bloom (Figure S4). Strong positive correlations (*r *>* *0.6 on average) were present between single fruit weight, fruit size, core size, shape and roughness of fruit stem, while these traits had a negative correlation (*r *<* *−0.25) with the stone cell and fruit hardness (Figure S5; Table S8). In addition, there was significant positive correlation between early bloom and full bloom (*r *>* *0.76), as well as between stone cell and fruit hardness (*r *>* *0.38) (Figure S5; Table S8).

**Table 2:** Broad‐sense heritability of 14 fruit quality traits used in the genome‐wide association study

Traits	Plot basis	Family mean basis
FW	0.85	0.94
LDF	0.88	0.96
TDF	0.83	0.94
LDC	0.71	0.88
TDC	0.73	0.89
LFS	0.61	0.82
RFS	0.61	0.82
SS	0.79	0.92
HN	0.69	0.86
SUS	0.52	0.76
SC	0.93	0.97
TS	0.95	0.98
SSU	0.98	0.99
CS	0.80	0.92

CS, core size; FW, fruit weight; HN, hardness; LDC, longitudinal diameter of fruit core; LDF, longitudinal diameter of fruit; LFS, length of fruit stem; RFS, roughness of fruit stem; SC, stone cell; SS, sepat state; SSU, soluble sugar; SUS, soluble solid; TDC, transverse diameter of fruit core; TDF, transverse diameter of fruit; TS, total sugar.

**Figure 5:**
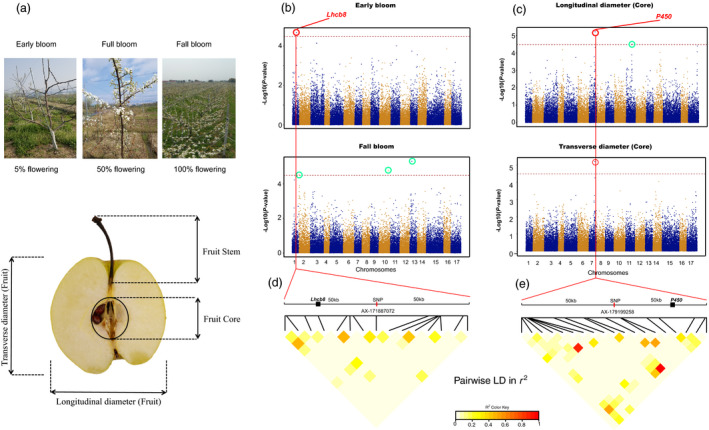
Genome‐wide association study (GWAS) results for 18 phenological and fruit quality traits from 219 pear accessions. (a) A schematic diagram of pear fruit and the investigation of phenological traits. (b) GWAS results for the early bloom and fall bloom. (c) GWAS results for the longitudinal and transverse diameter of pear fruit core. The red dashed horizontal line represents a significant threshold level (3.23 × 10^−5^). The SNPs in red and blue circles represent significant marker‐trait association loci, and important genes detected in their flanking region. Other significant loci are in green circles. (d) LD heatmap for the flanking 50 kb of significant SNP AX‐171887072. (e) LD heatmap for the flanking 50 kb of significant SNP AX‐1791997258.

The phenotypic data (Figure 5a) of the 18 traits were used along with genotypic data from 200K AXIOM^®^ PyrSNP array to perform GWAS. Results showed that nine of 18 trait associations identified a total of 42 significant (*P*‐value < 3.23 × 10^−5^) loci (Table S9), where at least one significant marker showed association in a trait. No significant marker was identified from the association analysis of fruit size, germination, fruit weight, total sugar, sepal state, soluble sugar, soluble solid and fruit core size. The significant SNP associated regions in the remaining traits were further analyzed for candidate genes. We identified 346 candidate unigenes associated with nine traits (Table S10). An SNP marker significantly (*P* = 2.08 × 10^−5^) linked with early bloom showed association with a known flowering gene (Figure 5b; Table S11). This marker was flanked by a homolog of light‐harvesting complex II protein *Lhcb8* (*Pbr009517.1*) (Figure 5b; Table S11). In addition, three SNPs, with *P*‐values as low as 6.09 × 10^−6^, showed a significant marker‐trait association with the longitudinal and transverse diameter of pear fruit core. A total of 26 genes were detected in the 50 kb flanking region of the three significant SNPs. Among them, 13 genes have been annotated for function in other species. For example, *Pbr002925.1* gene on chromosome 7 is homologous to *P450* (Figure 5c; Table S12), a gene that participates in the regulation of seed size in higher plants (Ito and Meyerowitz, 2000; Ma *et al*., 2015; Tian *et al*., 2016; Winkler and Helentjaris, 1995). The remaining 13 genes currently lack annotations.

## Discussion

A diverse SNP discovery panel and the inclusion of SNPs from Asian and European pears make 200K AXIOM^®^ PyrSNP array ideal for genetic studies, genomic selection and breeding of pear worldwide. The array has shown its usefulness and robustness in providing a large number of markers for dense genetic map construction and for identifying marker‐trait associations in several desirable pear traits. Such high‐density and genome‐wide coverage of SNP markers in 200K AXIOM^®^ PyrSNP array can provide opportunities to fine map genetic loci associated with several economical traits in different pear genetic backgrounds.

Asian pear has higher genetic diversity and a more complicated genetic than European pear (Wu *et al*., 2018), which likely explains the differences in the number of SNPs between Asian and European groups under same filtering criteria. There were 17 895 SNPs common among all possible pairs of the four groups with more SNPs common among Asian groups and European groups, than between Asian and European groups. A large number of unique SNPs within each group that distinguish it from the others represents the genetic diversity, genetic distance and characteristics of each group itself. Considering the well‐established genetic divergence between Asian and European pear and genomic differences in wild and domesticated pear within each group (Wu *et al*., 2018), an integration of SNPs from these four diverse groups might provide comparatively more genotyping coverage and reliability than the reference genome‐based marker discovery and genotyping. SNPs from both the European and Asian pear on this array will likely overcome some of the limitations associated with genome level differences among these groups and will help avoid the ascertainment bias caused by the rare variants from diverse genetic backgrounds. For instance, partial alignments between European and Asian pear sequences can lead to significant loss of marker information from sequence‐based genotyping approaches (Wu *et al*., 2018). Thus, pear SNP array has extensive benefits for exploring broader germplasm collections including segregating and breeding populations both within and between Asian and European pear, without losing a significant amount of marker information.

The high proportion (36%) of MonoHighResolution SNPs compared to the rather limited proportion of PolyHighResolution (17%) and NoMinorHom (16%) was likely due to the population group used to validate the array in the study and its size. The SNPs designed on 200K Axiom^®^ array were selected from four different pear groups i.e. Asian cultivated, Asian wild, European cultivated and European wild. The population genotyped to validate the 200K Axiom^®^ array mainly included Asian cultivated pears and their hybrids. The 200K SNPs that did not show polymorphism in this group potentially have polymorphism in another of the three groups. In apple, a total of 1324 apple accessions were genotyped to validate the Axiom^®^ Apple480K SNP array. PolyHighResolution SNPs presented the highest proportion (80%), likely due to the large population used (Bianco *et al*., 2016). In wheat, 475 accessions were genotyped using the Axiom^®^ Wheat HD Genotyping Arrays. A higher proportion (17.6%) of MonoHighResolution was seen, compared to the PolyHighResolution category (6.5%). Recently, development and validation of a 70K Affymetrix Axiom Genotyping Array for pear was presented at Plant and Animal Genome XXVI conference and 9th International Rosaceae Genomics Conference (RGC9) without data publication (Montanari *et al*., 2018). The array is based on re‐sequencing data of 55 accessions and showed more than 90% of the SNPs as high quality and polymorphic (PolyHighResolution) on The National Clonal Germplasm Repository pear accessions.

Use of dense genetic maps to improve the quality of a genome assembly has been widely applied across plant species (Bianco *et al*., 2016; Lee *et al*., 2015). Likewise, the high density and uniform distribution of SNPs on the pear array led to improvement in the current pear genome assembly by adding previously unanchored scaffolds. A high‐density genetic map was developed and used to assign chromosomal positions for many unanchored sequences in the current pear genome. The rate of genome assembly of pear was improved by 5.9%. However, completion of the pear genome assembly by placing remaining unanchored scaffolds on chromosomes will still require more genotyping and genetic mapping from diverse genetic backgrounds. The pear SNP array further guided the prediction of the centromeric regions of 17 pear chromosomes. The centromeric regions were lacking in the current version of pear genome (Wu *et al*., 2018) and relative SNP distribution and spacing was used as a measure to identify potential centromeric regions on chromosomes. The centromeric regions are complex due to the presence of a high amount of repetitive sequences, which usually cause suppression of recombination and low SNP density across these regions (Daccord *et al*., 2017; Lee *et al*., 2013; Chen *et al*., 2002; Saintenac *et al*., 2009; Schnable *et al*., 2009). Several low‐density SNP regions and large gaps between adjacent SNPs indicated the putative localization of centromeric regions on different pear chromosomes. However, a more rigorous analysis of genome‐wide recombination patterns can precisely define the centromeric and pericentromeric boundaries across the pear genome.

The array was also useful for assessing genotypic diversity and further clarified genetic relationships among 286 pear accessions. PCA clearly separated 286 pear accessions into two independent groups, with a higher genetic diversity in Group I than Group II. Similar results from population structure analysis further supported the diverse genetic background of Group I. The extensive germplasm collection contained within Group I pretty much explain this pattern. In contrast, Group II represented the hybrid progeny of a bi‐parental cross between ‘Cuiguan’ × ‘Starkrimson’, with limited diversity defined by the genomic composition of the two parental accessions. Most of the individuals in F_1_ progeny were clustered together to constitute Group II with approximately equal distance from the female parent ‘Cuiguan’ and the male parent ‘Starkrimson’. This suggests that genomes from both parental genotypes were equally represented in the F1 progeny. This observation was further supported by the large number of markers with 1:1 segregation. F1 individuals that grouped with the Group I cluster may be the result of outcrossing with an Asian pollen donor. It is to be expected in cross‐pollinated species that some level contamination by foreign pollen can occur. Even though we used the strict pollination methods, emasculation and then bagging, to control the crossing in our study, we also cannot completely rule out the possibility of outcrossing probably due to pollen contamination from Asian accession.

Identification of loci or genes that control high‐value traits is the main goal of pear genetics, breeding and improvement efforts. We used the pear array to perform GWAS and identify significant marker‐trait associations for 19 phenological and fruit traits. Significant associations were found for many traits at *P*‐value as low as 3.23 × 10^−5^. Several significant SNP associations were still apparent after lowering the stringency criteria to the top 0.1% or 1%. For example, fruit size is a highly complex trait that is controlled by a relatively small effect loci (Frary *et al*., 2000). Multiple loci associated (*P*‐value of 6.09 × 10^−6^ and 6.21 × 10^−6^) with the longitudinal and transverse fruit core diameter have been detected in this study. These loci correspond to candidate genes with homologs related to seed size in other plant species (Devoghalaere *et al*., 2012; Ma *et al*., 2015; Tian *et al*., 2016). Fruit core size has a direct impact on edible rate; thus, these genes can be explored further to improve productivity and fruit quality in pear. Similarly, flowering time is a fundamental trait required for pear adaptation to different climatic conditions. Although this trait has been extensively studied in other crops, genetic analysis of flowering time control in pear is still limited. We have identified one gene associated (*P*‐value of 2.08 × 10^−5^) with early bloom in pear. It is a homolog of *Lhcb8*, a gene that participates in light harvesting and transfer in high plants. It collects and transfers light energy to the reaction centres of photosystem II and controls plant flowering (Teramoto *et al*., 2001). We imposed a strict linkage disequilibrium (LD) based criteria to define the flanking regions and search candidate genes around significant marker‐trait associations detected in this study. LD decays rapidly in pear due to high heterozygosity and self‐incompatibility (Wu *et al*., 2018). Therefore, the number of candidate genes identified in this study is relatively low. Yao *et al*. (2017) used longer flanking sequences (150 kb) to search the candidate genes surrounding a significant QTL. Wider flanking intervals can show more candidate genes associated with each trait of interest and will require additional efforts for functional validation of these candidate genes.

In summary, we developed and validated the first high‐density and diverse SNP array of pear, and further demonstrated its broad applications for genotyping, genetic map construction, improving genome assembly and performing GWAS in pear. The large SNP dataset used to develop 200K AXIOM^®^ PyrSNP array, including the validated trait‐linked SNPs and SNPs from the whole genome re‐sequencing analysis of worldwide pear accessions, makes it ideal for genetic studies for both Asian and European pear. Uniform distribution of a large number of SNPs across 17 chromosomes of pear suggests that the array can be used effectively for genetic mapping of high‐value traits and identification of candidate genes through GWAS. This array will serve as a low cost, time sensitive and high‐throughput genotyping resource for genetic mapping, germplasm evaluation and genomic selection in pear.

## Materials and methods

### Re‐sequencing SNP discovery panel

High‐depth genome re‐sequencing data generated by Wu *et al*. (2018) were used in this study. A total of 113 pear accessions from 33 *Pyrus* species (22 well recognized pear species and other 11 species with separate Latin name named by collectors in different counties) (Wu *et al*., 2018), including 31 Asian cultivated, 32 Asian wild, 25 European cultivated and 25 European wild genotypes originating from 26 countries were analyzed for SNP discovery (Table S1). These accessions have all five major cultivated pear species, most recognized wild species and some local ecotypes representing global geographic distribution and genetic diversity in pear. Genomic DNA was extracted from frozen young leaves of 113 accessions using the CTAB method (Doyle and Doyle, 1987). Paired‐end DNA sequencing libraries with ~500 bp of short inserts were constructed and sequenced on HiSeq™ 2000 or HiSeq™ 4000 platforms (Illumina, San Diego, CA). Raw sequencing reads with fewer than 5% missing (*N*) bases and fewer than 50% of bases with a quality score <5 were retained and used for alignment and SNP detection in later steps (Wu *et al*., 2018).

### SNP detection, SNP selection and array development

The methods of SNP calling have been described in previous study (Wu *et al*., 2018). More than 18.3 million high‐quality SNPs from the previous whole‐genome re‐sequencing analysis were identified and used to design the 200K AXIOM^®^ PyrSNP array. An initial SNP quality analysis of the array was performed using following criteria: minor allele frequency (MAF) ≥ 0.1, missing genotypes <10% and LD *r* ^*2*^ < 0.4 based on the four groups (Asian wild, Asian cultivated, European wild and European cultivated groups). A set of 222 526 quality filtered SNPs from 17 chromosomes and another set of 44 001 SNPs from unanchored scaffolds were selected. These SNPs were primarily selected from the coding sequences (CDS) of genes identified genome‐wide. Additionally, 29 298 SNPs from the CDS regions of 1204 genes annotated as the trait‐associated genes by Wu *et al*. (2013), 3452 SNPs from the CDS regions of 240 genes reported from 48 literatures, 390 SNPs associated with 14 traits from GWAS analysis and 2059 SNPs on the 18 genes associated with sugar synthesis (Li *et al*., 2015) were included. A list of 299 109 candidate SNP loci was sent to the Affymetrix Bioinformatics Services (Santa Clara, CA) for final selection and the design of the array. Quality of each SNP was assessed and designated as ‘recommended’, ‘neutral’, ‘not_recommended’ and ‘not_possible’ using an *in silico* validation with a proprietary software. We retained one SNP marker for every 200 bp to ensure uniform distribution and high density of SNPs throughout the pear genome. The final 200K AXIOM^®^ PyrSNP genotyping array has a total of 200 481 SNP markers.

### Validation of 200K AXIOM^®^ PyrSNP array

A panel of 188 diverse pear accessions (Table S3) and an F_1_ progeny of ‘Cuiguan’ × ‘Starkrimson’ cross (98 individuals) were genotyped for the initial validation of the 200K AXIOM^®^ PyrSNP array. In F_1_ progeny, maternal parent ‘Cuiguan’ is an Asian cultivar pear (*P. pyrifolia*); while male parent ‘Starkrimson’ is a European cultivated pear (*P. communis*). It has been reported that both Asian and European species are distantly related but underwent independent domestication (Wu *et al*., 2018). Genomic DNA was extracted from frozen young leaves using the TIANGEN extraction kit (Tiangen Biotech Co., Ltd., Beijing, China). For each sample, 1 μg of genomic DNA was used, and 260 ng DNA of 20 ng/μL concentration with molecular weight ≥10 kb, OD260/280 range 1.7–2.1 was hybridized to the 200K SNP array by CapitalBio Technology (Beijing, China, http://www.capitalbiotech.com). The sample quality was controlled using both the value of Dish Quality Control (DQC) and sample call rate in the Affymetrix^®^ Power Tools (APT) software package. Briefly, the QC and genotype calls were generated from the hybridization signals from the CEL files created by the GeneTitan Multi‐Channel Instrument. The DQC value is the intensity contrast between signal and noise as measured by non‐polymorphic probes. A value of 1 for DQC indicates perfect resolution. In general, high DQC value represents the high call rate. However, sample call rate must be considered along with DQC values since the latter are insensitive to sample and DNA contamination. All accessions genotyped on the array were analyzed using Axiom Analysis Suite v.1.1.1 (Affymetrix, Waltham, MA) using diploid threshold configurations. In addition, two duplicated samples were included in each 96‐well plate for analysis of data reproducibility. Each SNP was classified into one of six categories with gradual reduction in reliability using ‘SNPolisher’ R Package designed by Affymetrix (http://www.affymetrix.com). The six categories are: ‘PolyHighResolution’, ‘NoMinorHom’, ‘OTV’, ‘MonoHighResolution’, ‘CallRateBelowThreshold’ and ‘Other’. ‘PolyHighResolution’ is the most reliable category for which the SNP passes all quality control criteria, including call rate (CR) ≥97%, Fisher's linear discriminant (FLD) ≥3.6, heterozygous strength offset (HetSO) ≥−0.1 and homozygote ratio offset (HomRO) values ≥0.3, whereas SNPs in other categories pass only some of these criteria. The CR value is the ratio of the number of genotyped samples against the number of expected samples for a probe. The FLD value is used to measure the cluster quality of a SNP based on the distance between the clusters and the variance within a cluster. The HetSO value measures the distance of the heterozygous cluster centre above or below from the homozygous cluster centres in the *y* dimension. Low HetSO values result from mis‐clustering events or inclusion of samples that contain variation from the reference genome that was used to design the array probe. The HomRO value is the distance to zero in the *x* dimension from the centre of the homozygous cluster. It indicates the shift of clusters from their expected positions. SNPs identified as ‘Other’ category were excluded from further analysis. The remaining SNPs were annotated using in‐house python script, and retained for further analyses. SNP count and distribution patterns across the pear genome were analyzed using a 150 kb sliding window to predict centromeric regions on pear chromosomes using R package.

### Population structure analysis

We converted the VCF file to GENO using an in‐house python script, and then used the LEA package (http://membres-timc.imag.fr/Olivier.Francois/tutoRstructure.pdf) in R software to calculate the population structure with default parameters: maximum number of iterations, 200; regularization parameter, 100; and tolerance error, 1e‐5. The number of clusters (*K*) was set from 2–4. Population structure was displayed using barplot() function in R package. Principal component analysis was performed using PLINK V1.90 (Purcell *et al*., 2007), VCFtools V0.1.13 (http://vcftools.sourceforge.net/man_latest.html) and Genome‐wide Complex Trait Analysis (GCTA) version 1.26.0 (Yang *et al*., 2011). The figure was plotted using ggplot2 (https://cran.r-project.org/web/packages/ggplot2/) package in R software. We used SNPhylo (Lee *et al*., 2014) to construct an ML tree with 1000 bootstraps, and displayed it using Figtree (http://tree.bio.ed.ac.uk/software/figtree/). All analyses were performed using the 166 335 high‐quality SNPs, and MAF ≥5% and missing rates <0.2 was used to further filter this SNP set.

### Linkage map construction

A segregating F_1_ population of ‘Cuiguan’ × ‘Starkrimson’ was used for genetic linkage map construction. The calls for high‐quality SNPs (66 214 SNPs), only included ‘PolyHighResolution’ and ‘NoMinorHom’ categories, were converted into the marker code system of three segregation types, ‘lmxll’, ‘nnxnp’ and ‘hkxhk’ as described in the cross‐pollinated (CP) model of JoinMap v4.1 software (Voorrips *et al*., 2006). The marker code ‘lmxll’ represents markers with first parent heterozygous and second parent homozygous, ‘nnxnp’ represents markers with first parent homozygous and second parent heterozygous, and ‘hkxhk’ represents markers with both parents heterozygous. A chi‐square test was performed to test the segregation ratios of each marker. Significantly distorted markers (chi‐square threshold of 0.01) were excluded from further analysis. In addition, loci with more than 10 missing genotypes were filtered out. The remaining 7173 markers were used to construct a linkage map with ‘regression mapping’ algorithm and ‘kosambi’ mapping function in JoinMap v4.1. The minimum LOD (logarithm of odds) score of 4.0 was set to establish the 17 LGs. Each LG was named to correspond an individual chromosome of the pear reference genome ‘Dangshansuli’. The linkage map figure was generated using the plot() function in R software.

### Evaluation of phenological and fruit traits

Phenotypic data was collected for 18 phenological and fruit traits from 286 pear accessions in 2016 and 2017. The pear trees were planted in the experimental orchard of the College of Horticulture at Nanjing Agricultural University. Most grown for about 10 years in an orchard with minimal management. Three to six biological replicates of fruits from each genotype were collected at the maturation stage for phenotypic evaluation and quality assessment. A Vernier caliper was used to measure the longitudinal and transverse diameter (unit: cm) of fruit core and periphery. An electronic balance was used to measure the single fruit weight (unit: g). Digital display Atago PAL‐1 was used to measure the soluble solid content in fruit flesh. Fruit hardness was measured using hardness tester CT3‐10K (unit: g) (Zhang *et al*., 2015). Fruit shape was recorded as: 1—oblateness; 2—round; 3—oblong; 4—ovoid; 5—obovate; 6—conical; 7—cylindrical; 8—Spindle; 9—thin‐neck gourd‐shape; 10—gourd‐shape; 11—thick‐neck gourd shape. The different sepal states were recorded using Arabic numerals 1–3 as indicated in (Cao *et al*., 2006). Stone cell content (mg/g) was measured through the combined method of HCl separation and freeze processing (Tao *et al*., 2009). High‐performance liquid chromatography (HPLC, Waters 1525 HPLC system) was used to evaluate the soluble sugars (mg/g) per unit fresh weight (FW) (Liu *et al*., 2016). The dates for phenological periods, including germination, early bloom, full bloom and fall bloom stages were surveyed and recorded in the field every 2 days (Cao *et al*., 2006). Statistical analyses, including normal distribution, boxplot and Pearson correlation, were performed for 18 traits in R 3.2.0 (R Development Core Team, 2013) software (http://www.R-project.org). The outliers were checked for each trait individually using boxplot charts. Trait correlations were calculated and plotted in using cor() and heatmap.2() in gplots package (Warnes *et al*., 2012). Histograms were created using the hist() function, and boxplot charts were plotted using the boxplot() function. Variance components were used to estimate broad‐sense heritability as the ratio between the genotypic and the phenotypic variances: H2 = $\sigma_{g}^{2}/\sigma_{p}^{2}$. Whereas $\sigma_{p}^{2}$ is phenotypic variance calculated as ($\sigma_{g}^{2}$ + $\sigma_{e}^{2}$/*n*), $\sigma_{g}^{2}$ is the genotypic variance, $\sigma_{e}^{2}$ is the environmental variance and *n* the mean number of replicate per genotype.

### Genome‐wide association study

The mean phenotypic values per genotype for each of 18 phenological and fruit quality traits collected over 2 years (2016 and 2017) and total 31 388 SNPs with a MAF ≥5% from 286 pear accessions were used for GWAS. Individuals with 100% missing trait data were removed for further analysis. Genomic Association and Prediction Integrated Tool (GAPIT), an R package developed by Lipka *et al*. (2012), with a compressed mixed linear model was used to perform GWAS. The population structure as assessed by PCA and the relatedness calculated by the VanRaden method were used as co‐factors in the analysis. The final Manhattan plots were created using the ggplot2 package in R. The threshold for significant association was set to 1/*n* (*n* is the total marker numbers for all samples, *P *<* *3.23 × 10^−5^) (Wang *et al*., 2015; Yang *et al*., 2014). Candidate genes underlying significant SNP‐trait associations for each trait were identified from Asian pear genome ‘Dangshansuli’ by searching the nearby genes within 50 kb upstream and downstream of identified SNPs. Afterwards, genes underlying significant SNP regions were annotated to identify function of the candidate genes.

## Author contributions

X.L.L. carried out data analysis, prepared the figures and drafted the manuscript. J.S. provided support for data analysis, interpretation of results and manuscript writing. M.F.Q. constructed the genetic linkage map. S.W.L. and X.Z. collected samples and performed the experiments to measure the physiological data of all the samples. M.Y.Z. discussed the results from GWAS. A.K. helped with the interpretation of results and writing manuscript. J.W. and S.L.Z managed and designed the research.

## Competing financial interests

The authors declare no competing financial interests.

## Supporting information


**Figure S1** The distribution of 200 481 SNPs selected for pear array design on the physical maps of the 17 pear chromosomes.

**Figure S2** Population structure analysis of 188 accessions from Group I.

**Figure S3** Distribution of the converted SNPs on the array in sliding 150 kb windows along the each of 17 pear chromosomes.

**Figure S4** The normal distribution (blue bar) and boxplot charts (orange box) of phenotypic data from 18 traits.

**Figure S5** Correlation between 18 fruit quality and phenological traits assessed in a 188 diverse accessions of Asian pear.

**Table S1** The origin of sequencing 113 pear accessions.

**Table S2** List of the 200 481 SNP markers that were put on the array.

**Table S3** The origin of 188 pear accessions.

**Table S4** The details of 2388 SNPs mapped on the 17 linkage groups.

**Table S5** The consistency between the order of markers on the linkage map and genome sequence assembly.

**Table S6** The summary of 355 SNPs mapped the unanchored scaffolds on each linkage group.

**Table S7** The 18 imputed phenotypic data from 219 pear accessions.

**Table S8** The Pearson correlation of 18 traits from 219 accessions.

**Table S9** The information of 42 SNP markers significantly associated with nine traits.

**Table S10** The candidate genes in the flanking region of significant SNP markers associated with 12 traits.

**Table S11** The candidate genes in the flanking region of significant SNP markers associated with early bloom and their annotation information.

**Table S12** The candidate genes in the flanking region of significant SNP markers associated with core size and their annotation information.
