# Supplementary material for: Development of an integrated 200K SNP genotyping array and application for genetic mapping, genome assembly improvement and genome wide association studies in pear (Pyrus)
Source: Plant Biotechnol J. 2019 Feb 17;17(8):1582–94. doi: 10.1111/pbi.13085 (PMC6662108; doi:10.1111/pbi.13085)
Supplement: Supplementary file 1 — Figure S1 The distribution of 200 481 SNPs selected for pear array design on the physical maps of the 17 pear chromosomes. [file PBI-17-1582-s015.pdf]

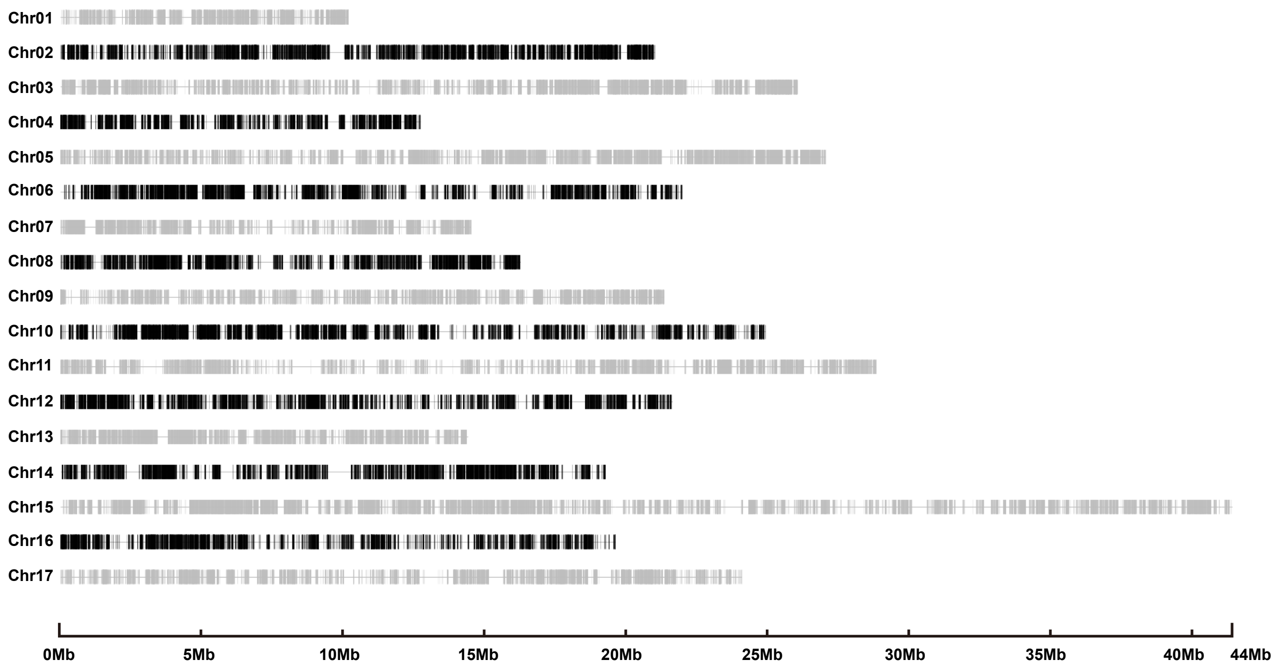

Figure S1 The distribution of 200,481 SNPs selected for pear array design on the physical maps of the 17 pear chromosomes (Chr). Each black and grey bar represents one SNP marker. The text on the left represents the chromosome number.
