# Supplementary material for: Development of an integrated 200K SNP genotyping array and application for genetic mapping, genome assembly improvement and genome wide association studies in pear (Pyrus)
Source: Plant Biotechnol J. 2019 Feb 17;17(8):1582–94. doi: 10.1111/pbi.13085 (PMC6662108; doi:10.1111/pbi.13085)
Supplement: Supplementary file 2 — Figure S2 Population structure analysis of 188 accessions from Group I. [file PBI-17-1582-s012.pdf]

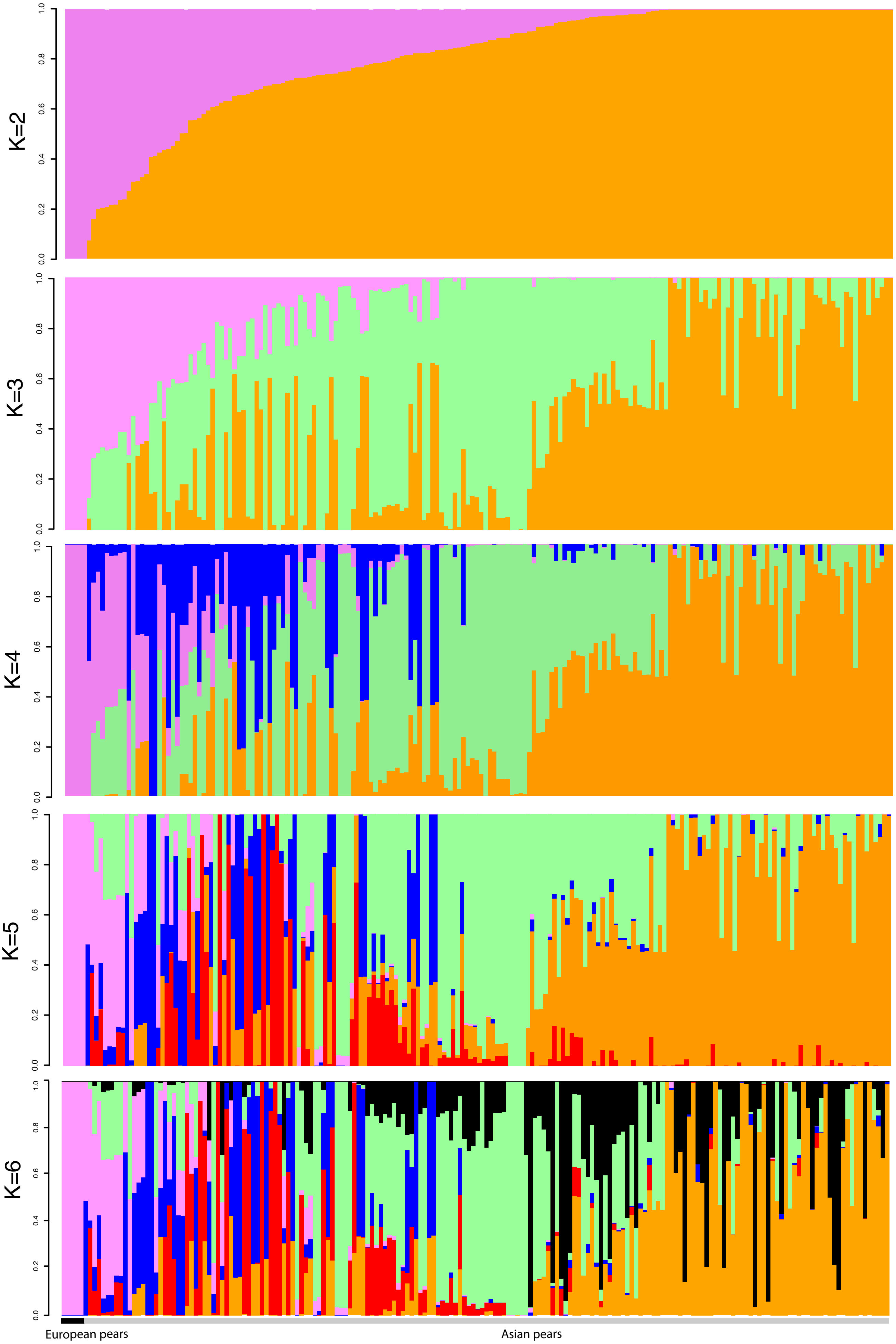

Figure S2 Population structure analysis of 188 accessions from Group I. The numbers of clusters (K) were set from 2-6. Each color represents a single population. Each bar represents one accession and different colored segment represents the proportion from ancestral populations.
