# Supplementary material for: Development of an integrated 200K SNP genotyping array and application for genetic mapping, genome assembly improvement and genome wide association studies in pear (Pyrus)
Source: Plant Biotechnol J. 2019 Feb 17;17(8):1582–94. doi: 10.1111/pbi.13085 (PMC6662108; doi:10.1111/pbi.13085)
Supplement: Supplementary file 3 — Figure S3 Distribution of the converted SNPs on the array in sliding 150 kb windows along the each of 17 pear chromosomes. [file PBI-17-1582-s001.pdf]

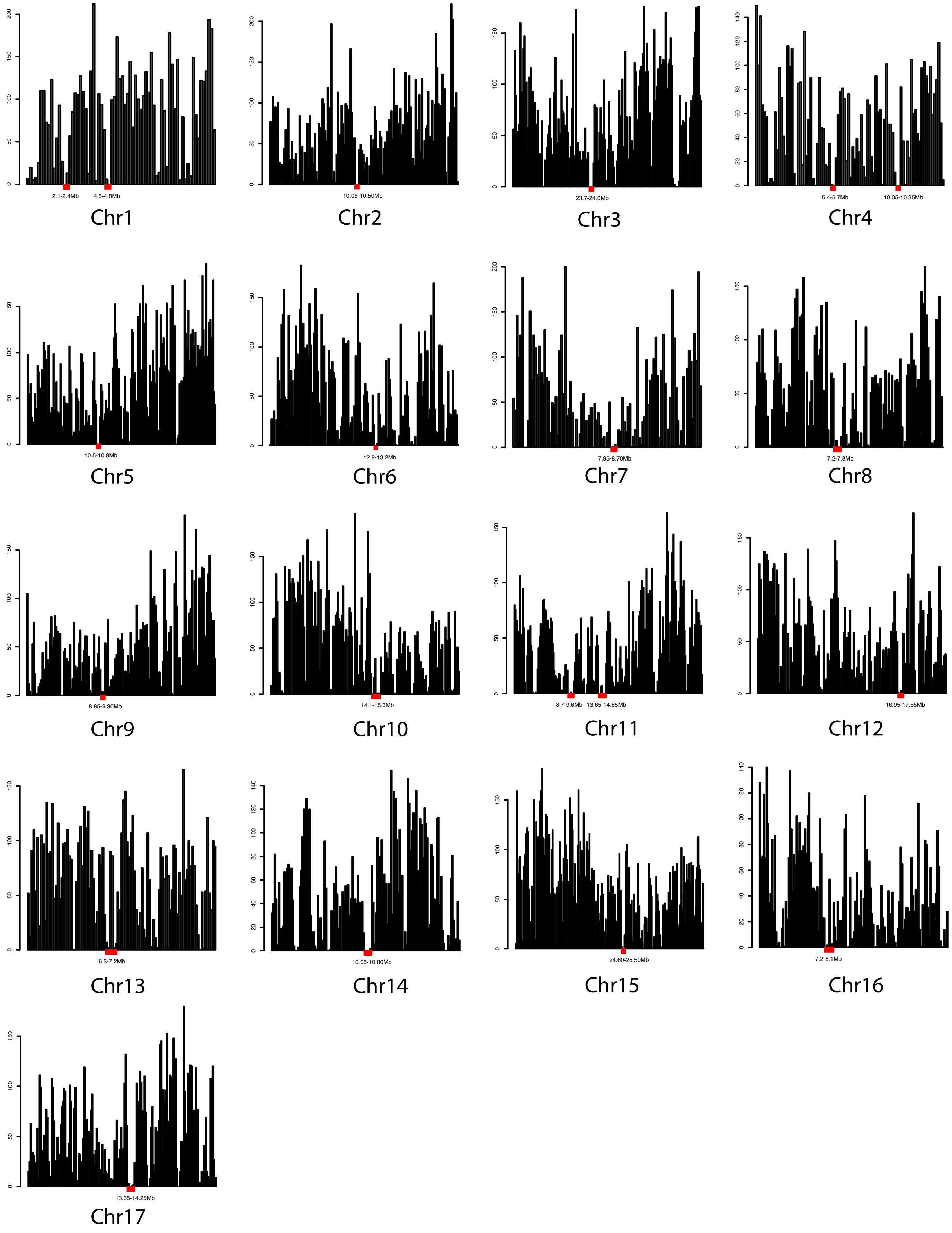

Figure S3 Distribution of the converted SNPs on the array in 150 kb sliding windows along the each of 17 pear chromosomes (Chr). Approximate centromere positions are indicated by the red dots on the x axis.
