# Supplementary material for: Development of an integrated 200K SNP genotyping array and application for genetic mapping, genome assembly improvement and genome wide association studies in pear (Pyrus)
Source: Plant Biotechnol J. 2019 Feb 17;17(8):1582–94. doi: 10.1111/pbi.13085 (PMC6662108; doi:10.1111/pbi.13085)
Supplement: Supplementary file 4 — Figure S4 The normal distribution (blue bar) and boxplot charts (orange box) of phenotypic data from 18 traits. [file PBI-17-1582-s016.pdf]

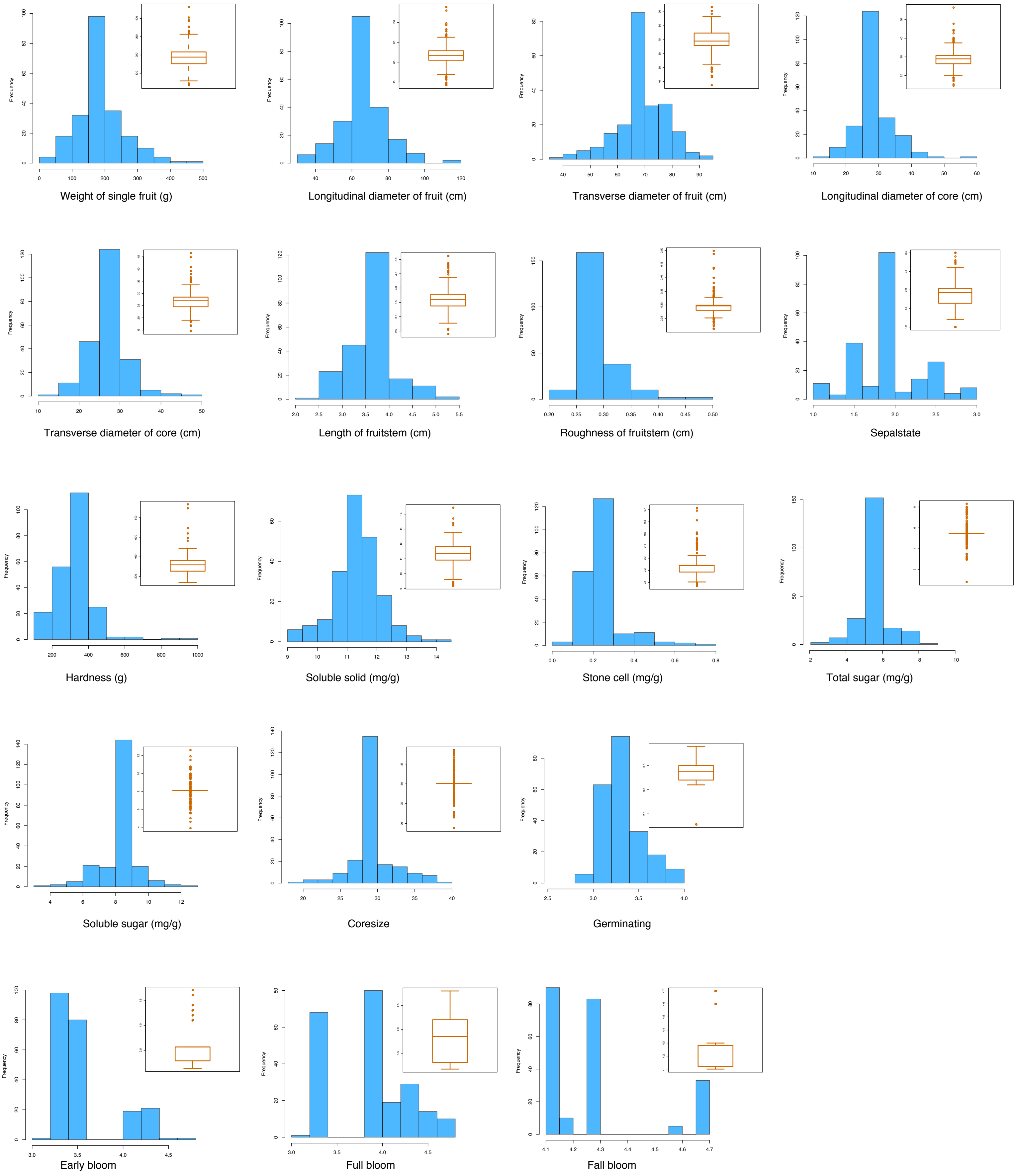

Figure S4 The normal distribution (blue bar) and boxplot charts (orange box) of phenotypic data from 18 traits.
