# Supplementary material for: Development of an integrated 200K SNP genotyping array and application for genetic mapping, genome assembly improvement and genome wide association studies in pear (Pyrus)
Source: Plant Biotechnol J. 2019 Feb 17;17(8):1582–94. doi: 10.1111/pbi.13085 (PMC6662108; doi:10.1111/pbi.13085)
Supplement: Supplementary file 5 — Figure S5 Correlation between 18 fruit quality and phenological traits assessed in a 188 diverse accessions of Asian pear. [file PBI-17-1582-s017.pdf]

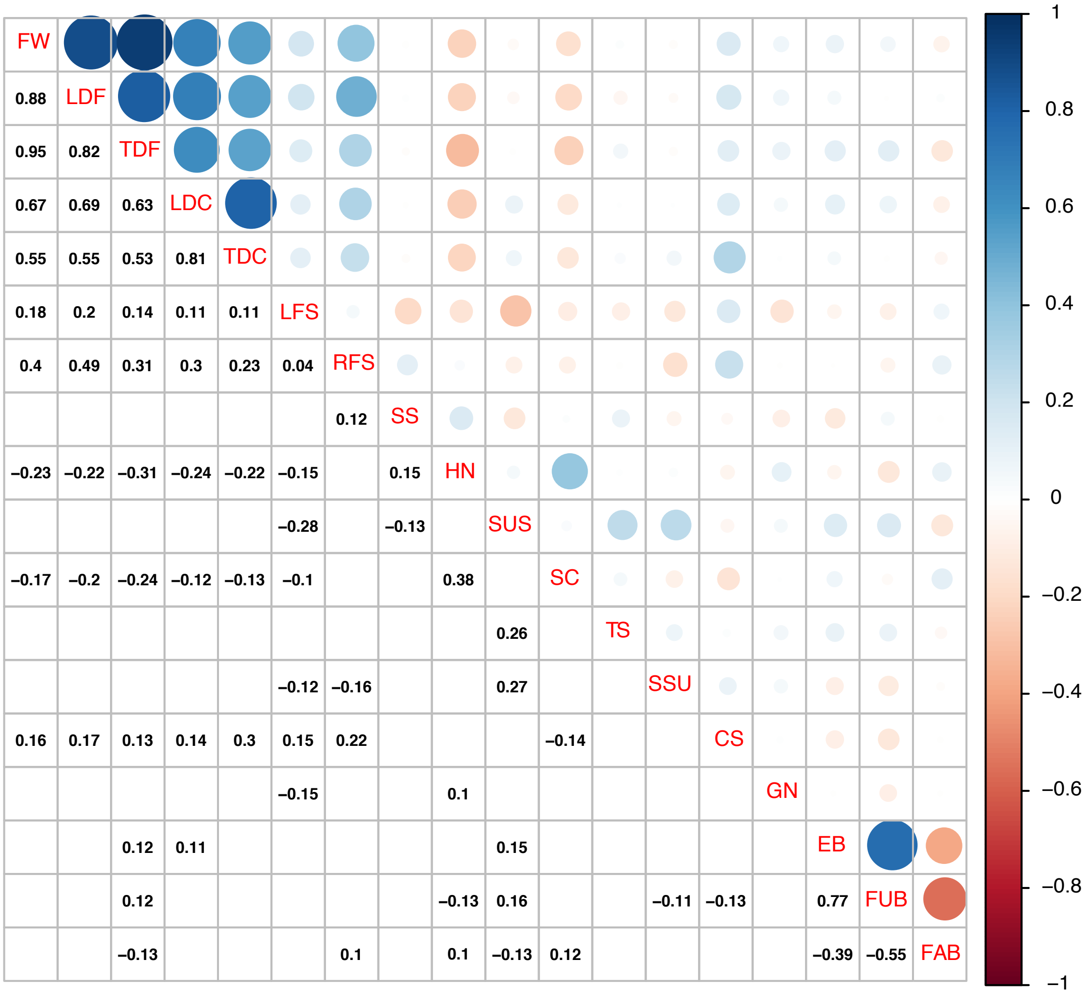

Figure S5 Correlation between 18 fruit quality and phenological traits assessed in a 188 diverse accessions of Asian pear. The color scale at the right represents Pearson correlation coefficient (PCC). Dark red indicates negative correlation and blue indicates positive correlation. FW, fruit weight; LDF, longitudinal diameter of fruit; TDF, transverse diameter of fruit; LDC, longitudinal diameter of fruit core; TDC, transverse diameter of fruit core; LFS, length of fruitstem; RFS, roughness of fruitstem; SS, sepatstate; HN, hardness; SUS, soluble solid; SC, stone cell; TS, total sugar; SSU, soluble sugar; CS, coresize; GN, germinating; EB, early bloom; FUB, full bloom; FAB, fall bloom. Cells with correlation values not significant at P-value < 0.01 are left blank.
